# Editorial: Coaching Systems for Health and Well-Being

**DOI:** 10.3389/fdgth.2021.658023

**Published:** 2021-02-25

**Authors:** Evdokimos I. Konstantinidis, Eleftheria Vellidou, Luis Fernandez-Luque, Panagiotis D. Bamidis

**Affiliations:** ^1^Laboratory of Medical Physics, School of Medicine, Aristotle University of Thessaloniki, Thessaloniki, Greece; ^2^WITA Srl, Trento, Italy; ^3^Biomedical Engineering Laboratory, Athens, Greece; ^4^Salumedia Labs, Seville, Spain

**Keywords:** virtual coach, coaching, health, well-being, personalized care, digital health, assistive technology, ambient intelligence

For years, there has been a call for the advancement of Digital Health (DH) as an enabler of the much-needed transformation of healthcare systems toward a more preventive and personalized paradigm. This transformation is, however, complex and does require a system-wide effort for the generation of evidence and best practices (1). A particular area of interest is the use of DH to support people living with chronic conditions, not only in terms of managing their diseases, but also improving both their physical and mental well-being. In this special issue, we explore new directions on digital health coaching systems for health and well-being.

Health coaching systems are readily available in the patients' environments. There is a rapid growth in the use of smartwatches and wearable technologies in general, besides the omnipresent mobile phones. The review of Sqalli and Al-Thani provides insights on how wearables can be used as coaching enablers, including examples of major chronic conditions and the prominent challenges that this type of technology currently faces. An example of how such coaching systems can be put in place is described by Tsakanikas et al. providing an evaluation of a physical rehabilitation coaching system that combines wearable technologies with virtual reality. The vCare project (Kyriazakos et al.), also with a focus on home-based rehabilitation, particularly highlights the importance of integrating such systems into the clinical pathways as a key aspect to consider in the implementation of DH coaching solutions.

Another important trend is the digitisation of homes, especially important for the development of smart homes supporting elderly citizens through the adoption of the Internet of Things (IoT) technologies. Palumbo et al. provide an example of how IoT can be used as the foundation of healthy aging coaching aiming at preventing frailty while promoting healthy aging. Another example of coaching technologies for healthy living is the promotion of healthy habits amongst children, which is covered in another study presented by Beun et al. where the focus is on the use of gamification techniques to support both user engagement and health behavioral change.

Over the last few years, radically new solutions for a personalized “virtual coach” have emerged with new ways of interaction based on tangible user interaction concepts (2). The feasibility studies and the positive evaluation of their acceptability as well as their effectiveness in changing user's behavior (3) will open a new era when virtual coaches will be considered as effective solutions for providing personalized advice, guidance and follow-up for persons in need of care. The more the virtual coaches are designed to empower users' by supporting their intrinsic desire for behavior change, the more effective they will be proven contrary to traditional systems instructing the users. Such forms of interaction, integrated seamlessly in existing every-day activities and providing desired information in fast and efficient manner, could contribute to physical, cognitive, mental and social well-being preservations.

The studies published in this special issue provide a window into some of the challenges that are going to be crucial in the short term, especially with regards to the recovery of the COVID-19 pandemic where the health of millions of people with chronic conditions has been negatively affected. The large scale and unprecedented adoption of digital health coaching systems will continue to create new opportunities and a need for research in this area. To conclude, it is important to stress one thing: Research efforts and projects should not only look into the effectiveness of coaching systems, but also innovative ways for the provision of training to healthcare professionals, as well as, (formal and informal) care provides, who are amongst the key stakeholders in ensuring the safe adoption of these new technologies.

## Author Contributions

EK, EV, LF-L, and PB contributed equally to the editorial. All authors contributed to the article and approved the submitted version.

## Conflict of Interest

EK was employed by WITA srl. LF-L is General Manager and founder at Salumedia Labs SLU (Spain) and Chief Scientific Officer at Adhera Health Inc (USA). The remaining authors declare that the research was conducted in the absence of any commercial or financial relationships that could be construed as a potential conflict of interest.
